# Immunological and clinical characteristics of severe thrombocytopenia in neonates with Kasabach–Merritt phenomenon

**DOI:** 10.3389/fimmu.2026.1847882

**Published:** 2026-06-29

**Authors:** Cancan Li, Yang Li, Yan Huang, Yuanyuan Gao, Lili Li, Jinhui Hu, Ting Feng, Zhenhong Zhu, Haifeng Geng

**Affiliations:** 1 Department of Neonatology, Children’s Hospital of Soochow University, Suzhou, China; 2 Department of Clinical Laboratory, Children’s Hospital of Soochow University, Suzhou, China; 3 Department of Pharmacy, Children’s Hospital of Soochow University, Suzhou, China; 4 Department of Neonatology, The Affiliated Suzhou Hospital of Nanjing Medical University, Suzhou, China; 5 Neonatal Medical Center, Huai’ an Maternity and Child Health Care Hospital, Huaian, China; 6 Department of Ultrasonography, Children’s Hospital of Soochow University, Suzhou, China; 7 Department of Burn and Plastic Surgery, Children’s Hospital of Soochow University, Suzhou, China

**Keywords:** clinical characteristics, hemangioma, immunological features, Kasabach-Merritt phenomenon, neonates, severe thrombocytopenia

## Abstract

**Objective:**

This study aimed to characterize the immune profile of neonates with Kasabach-Merritt phenomenon (KMP) and to identify clinical features linked to severe thrombocytopenia in these patients.

**Methods:**

This multicenter retrospective study included neonates diagnosed with KMP based on established criteria. The peripheral blood lymphocyte subsets, complement levels, and humoral immunity were compared between KMP neonates and controls. KMP neonates were further divided into severe and non-severe groups based on whether the platelet count was <20 × 10^9^/L. Clinical data and treatment outcomes were compared between the two groups, and multivariate analysis was performed to identify independent factors associated with severe thrombocytopenia.

**Results:**

A total of 32 KMP neonates were included (13 in the severe group and 19 in the non-severe group). Compared with controls, KMP neonates had reduced CD3^+^CD4^+^ and CD3^−^CD19^+^ proportions and lower IgG levels (all *P* < 0.05). The most common type of KHE lesion in KMP neonates was superficial + mixed (24/32, 75.00%), with a median maximum diameter of 68.50 (52.50–87.25) mm and a platelet count of 41.50 (17.25–48.75) × 10^9^/L. Of the KMP neonates, 25 achieved complete remission (78.1%), 4 achieved partial remission (12.5%), and 3 had no remission (9.3%). Compared to the non-severe group, the severe KMP group had larger diameters. The severe group also had lower CD3+CD4+ proportions, and significantly higher levels of lymphocytes percentage (L%), PCT, CKMB, and ferritin, along with prolonged APTT (P < 0.05). No significant difference in prognosis was observed between the two groups (*P* > 0.05). Multivariate regression analysis identified prolonged APTT and elevated ferritin as independently associated with severe thrombocytopenia in KMP.

**Conclusion:**

Neonates with KMP showed immunological abnormalities, including decreases in helper T cells and B cells. Prolonged APTT and elevated ferritin were identified as independent risk factors for severe thrombocytopenia in KMP.

## 1 Introduction

Kasabach-Merritt phenomenon (KMP) is a rare but life-threatening coagulopathy associated with vascular tumors, commonly observed in infants and young children. According to the latest International Society for the Study of Vascular Anomalies (ISSVA) Classification and the recent international expert consensus statement (1, 2), KMP is now recognized as a phenomenon specifically associated with kaposiform hemangioendothelioma (KHE) and tufted angioma (TA), rather than a shared complication of multiple vascular tumors. The primary characteristics of KMP include severe thrombocytopenia as a core defining feature, along with consumptive coagulopathy, and hemorrhagic complications related to vascular tumors (3–5). Increasing evidence suggests that the immune system plays an active role in vascular abnormalities and vascular tumor-related diseases, not merely as a bystander. It may influence disease progression through involvement in inflammation regulation, angiogenesis, and coagulation processes (6, 7). Studies have pointed out that inflammation and changes in the immune microenvironment can affect endothelial cell function and vascular abnormalities (8). Additionally, in various immune-related diseases, the proportion and functional changes of immune cell subsets have been shown to correlate closely with disease activity and severity, indicating the potential value of dynamic immune changes in disease assessment (9, 10). However, research on KMP in children has mainly focused on clinical characteristics and treatment strategies, with limited systematic studies on the dynamic changes of immune cell subsets and humoral immune markers (11).

Severe thrombocytopenia is one of the hallmarks of KMP and typically presents with a substantial reduction in platelet count, sometimes dropping below 20 × 10^9^/L. As the associated vascular tumor advances, abnormal vascular channels within the lesion cause ongoing platelet trapping, activation, and consumption, which results in a progressive decrease in peripheral platelet counts (8). This severe thrombocytopenia not only reflects excessive platelet consumption but is often accompanied by hypofibrinogenemia and consumptive coagulopathy, significantly impairing hemostatic function and increasing the risk of spontaneous bleeding, persistent bleeding, and bleeding related to invasive procedures (3, 8). Previous studies have also suggested that platelet count levels are closely associated with disease severity. Persistent or progressive declines often indicate increased disease activity and may be related to prolonged disease duration and increased treatment complexity (1, 3, 12). Although thrombocytopenia is critical for the diagnosis and disease assessment of KMP, systematic research on the clinical characteristics and influencing factors of severe thrombocytopenia in KMP is still limited, and the underlying mechanisms remain to be further elucidated.

Accordingly, this study first focused on the immunological profile of neonates with KMP by systematically evaluating peripheral blood lymphocyte subsets and humoral immune markers, with the aim of shedding new light on the immune mechanisms underlying KMP. We further explored the clinical features and correlates of severe thrombocytopenia in KMP in neonates, hoping to provide a theoretical basis and evidence-based support for early recognition of severe thrombocytopenia, better risk stratification, and individualized treatment planning.

## 2 Methods

### 2.1 Study subjects and diagnosis

This study is a multicenter retrospective case-control study. Neonates diagnosed with KMP and treated between January 1, 2010, and December 31, 2024, at the Children’s Hospital of Soochow University, the Affiliated Suzhou Hospital of Nanjing Medical University, and Huai’ an Maternity and Child Health Care Hospital were included as study subjects.

The study was approved by the ethics committees of the hospital (Ethics No. 2025CS071). Written informed consent was obtained from the patient’s family, granting permission for the publication of their child’s clinical data. All methods were performed in accordance with the ethical standards outlined in the Declaration of Helsinki and its later amendments or comparable ethical standards.

### 2.2 Diagnostic and exclusion criteria

The diagnosis of KMP (3, 11) is based on significant thrombocytopenia and consumptive coagulopathy associated with invasive vascular tumors, supported by imaging evidence showing tumor characteristics, and excluding other causes of thrombocytopenia and coagulopathy. Specifically, the diagnosis was established through integrated clinical and radiological criteria, including (1): a rapidly enlarging violaceous indurated mass; (2) ultrasound and/or MRI demonstrating an infiltrative hypervascular lesion consistent with KHE or TA; and (3) laboratory findings of platelet count <100 × 10^9^/L, hypofibrinogenemia, and elevated D-dimer. Histopathological confirmation (spindle-shaped endothelial cells with D2–40 immunopositivity) was obtained in selected cases where biopsy was clinically feasible and the risk of hemorrhage was acceptable.

The exclusion criteria are: (1) a definitive diagnosis of genetic metabolic disorders or immunodeficiency; (2) primary hematologic disorders; (3) thrombocytopenia or coagulopathy caused by severe infections or other diseases; (4) thrombocytopenia attributable to vascular malformations, confirmed by imaging review to lack the solid infiltrative neoplastic features of KHE/TA; and (5) ordinary infantile hemangioma as the underlying lesion.

### 2.3 Grouping criteria

Neonates hospitalized during the same period, with an age difference of ±7 days, were selected as the control group at a 1:1 ratio. All control group neonates had no infections, immunological, or hematological disorders. Baseline characteristics, lymphocyte subsets, complement levels, and humoral immunity were compared between the two groups.

KMP patients were divided into severe and non-severe groups based on platelet count: those with platelet counts <20 × 10^9^/L were defined as the severe KMP group (n = 13), and those with platelet counts ≥20 × 10^9^/L were defined as the non-severe KMP group (n = 19). Clinical data of the two groups were compared.

### 2.4 Clinical data collection

Clinical data were extracted from the electronic medical records system and supplemented by telephone and outpatient follow-up. The main data collected included: (1) Baseline characteristics: gender, age at sampling, birth weight, gestational age, etc.; (2) Vascular tumor characteristics: type (superficial + mixed, deep, multifocal), color, and size. Specifically, lesion type was classified based on ultrasound and/or MRI findings. Superficial + mixed type was defined as lesions predominantly involving the skin and subcutaneous tissue, including lesions with both superficial and deep components when the major tumor burden remained within the superficial compartment. Deep type referred to lesions extending beyond the deep fascia with involvement of deep muscle, bone, or visceral organs. Multifocal type was defined as the presence of two or more anatomically distinct KHE/TA lesions without continuous imaging connection. For lesions involving both superficial and deep compartments within a single continuous mass, classification was determined according to the predominant compartment, defined as the layer containing the greatest tumor volume. Tumor color was graded using a standardized visual scale (red, purplish, or deep purple/ecchymotic) and independently evaluated by two clinicians, with discrepancies resolved by consensus. Tumor size was measured by ultrasound as the maximum diameter among three orthogonal dimensions and was recorded at baseline and follow-up using a standardized protocol.

(3) Immunological markers: lymphocyte subsets (CD3^+^, CD3^+^CD4^+^, CD3^+^CD8^+^, CD3^−^CD19^+^, CD3^−^CD16^+^56^+^, etc.), complement levels (C3, C4), and immunoglobulins (IgA, IgG, IgM); (4) Laboratory parameters included complete blood count (white blood cell count, hemoglobin, neutrophil/lymphocyte/monocyte/eosinophil/basophil/reticulocyte percentages); inflammatory markers (C-reactive protein [CRP] and procalcitonin [PCT]); myocardial injury markers (creatine kinase-MB [CK-MB] and high-sensitivity troponin); coagulation function (prothrombin time [PT], activated partial thromboplastin time [APTT], fibrinogen, D-dimer, and international normalized ratio [INR]); and biochemical parameters (total protein, albumin, globulin, total bilirubin, direct bilirubin, alanine aminotransferase [ALT], aspartate aminotransferase [AST], creatinine, urea, and ferritin); (5) Treatment and outcome indicators: corticosteroids, immunoglobulins, blood product transfusions, subsequent targeted therapy use, and short-term efficacy. Treatment was initiated in all patients meeting the diagnostic criteria for KMP. As this was a retrospective cohort from 2010 to 2024, treatment strategies followed the clinical practice at the time of management. Sirolimus was used in selected patients with refractory disease or severe KMP with active bleeding, with treatment indications retrospectively interpreted in light of the current international consensus statement (1). Recorded details included drug name, dose, and duration; transfusion type and frequency with specific indications; interventional procedures (embolization, surgery); and adverse events.

### 2.5 Efficacy assessment

Based on previously reported literature (11, 13, 14), the treatment outcomes of KMP in this study were defined as follows: (1) Complete remission (CR): complete disappearance of the lesion, with normalization of coagulation function and sustained platelet recovery to ≥100 × 10^9^/L on at least two consecutive assessments (2). Partial remission (PR): ≥25% reduction in lesion size on ultrasound or MRI compared with baseline, accompanied by normalization of coagulation function and stable platelet counts ≥50 × 10^9^/L (3). No response (NR): <25% reduction or enlargement of lesion size, persistent or worsening coagulation abnormalities, and platelet counts remaining <50 × 10^9^/L.

### 2.6 Statistical analysis

Data analysis was performed using SPSS version 27.0. For quantitative variables, data are presented as mean ± standard deviation (SD) or median (interquartile range), depending on the distribution of the data. The comparison between two groups was conducted using independent t-tests or Mann–Whitney U tests. For qualitative variables, data are expressed as frequencies and percentages, and group comparisons were performed using the chi-square test or Fisher’s exact test. Spearman correlation analysis was used to assess the correlations between key laboratory parameters, with |r| > 0.3 and a *P*-value < 0.05 considered statistically significant. Based on the results of univariate analysis, variables with significant differences were selected for multivariate logistic regression analysis. Given the collinearity of CK-MB, PCT, and CD3+CD4+, these were excluded, and lymphocytes percentage (L%), APTT, globulin, and ferritin were included as candidate variables in the multivariate regression model. The multivariate logistic regression model was constructed using stepwise regression, and binary logistic regression was used to assess factors associated with severe KMP. The model’s predictive performance was further validated using receiver operating characteristic (ROC) curves. All statistical tests were two-sided, with *P* < 0.05 considered statistically significant.

## 3 Results

### 3.1 Lymphocyte subsets and complement levels in neonates with KMP

The clinical baseline characteristics of neonates with KMP and the control group are detailed in **Table 1**. This study included 32 neonates with KMP and 32 controls. Among the KMP group, there were 15 male and 17 female neonates, with a gestational age of 38.57 (37.71–39.71) weeks and a birth weight of 3273.00 (2779.25–3676.00) g. No significant differences were observed between the two groups in terms of gender, birth weight, and gestational age (*P* > 0.05).

**Table 1:** Lymphocyte subsets and complement levels in neonates with KMP.

Variable	KMP group (n = 32)	Control group (n = 32)	P-value
Baseline characteristics			
Male, n (%)	15 (46.88%)	15 (46.88%)	1.000
Birth weight, g	3273.00 (2779.25-3676.00)	3250.00 (3045.00-3412.50)	0.732
Gestational age, weeks	38.57 (37.71-39.71)	39.00 (38.53-39.31)	0.397
Lymphocyte subsets			
CD3+	0.80 ± 0.06	0.78 ± 0.07	0.294
CD3+CD4+	0.53 ± 0.08	0.59 ± 0.06	0.003
CD3+CD8+	0.18 ± 0.03	0.20 ± 0.06	0.079
CD4+/CD8+	2.91 ± 0.71	3.18 ± 1.37	0.329
CD3-CD19+	0.08 ± 0.04	0.11 ± 0.05	0.012
CD3-CD(16 + 56)+	0.11 ± 0.06	0.10 ± 0.06	0.295
Complement and humoral immunity			
C3, g/L	0.71 ± 0.20	0.73 ± 0.17	0.559
C4, g/L	0.17 ± 0.07	0.18 ± 0.06	0.584
IgA, g/L	0.05 ± 0.02	0.05 ± 0.07	0.881
IgG, g/L	6.76 ± 1.54	7.74 ± 2.15	0.041
IgM, g/L	0.28 ± 0.13	0.28 ± 0.17	0.994

KMP, Kasabach–Merritt phenomenon; C3, complement component 3; C4, complement component 4; IgA, immunoglobulin A; IgG, immunoglobulin G; IgM, immunoglobulin.

**Figure 1:**
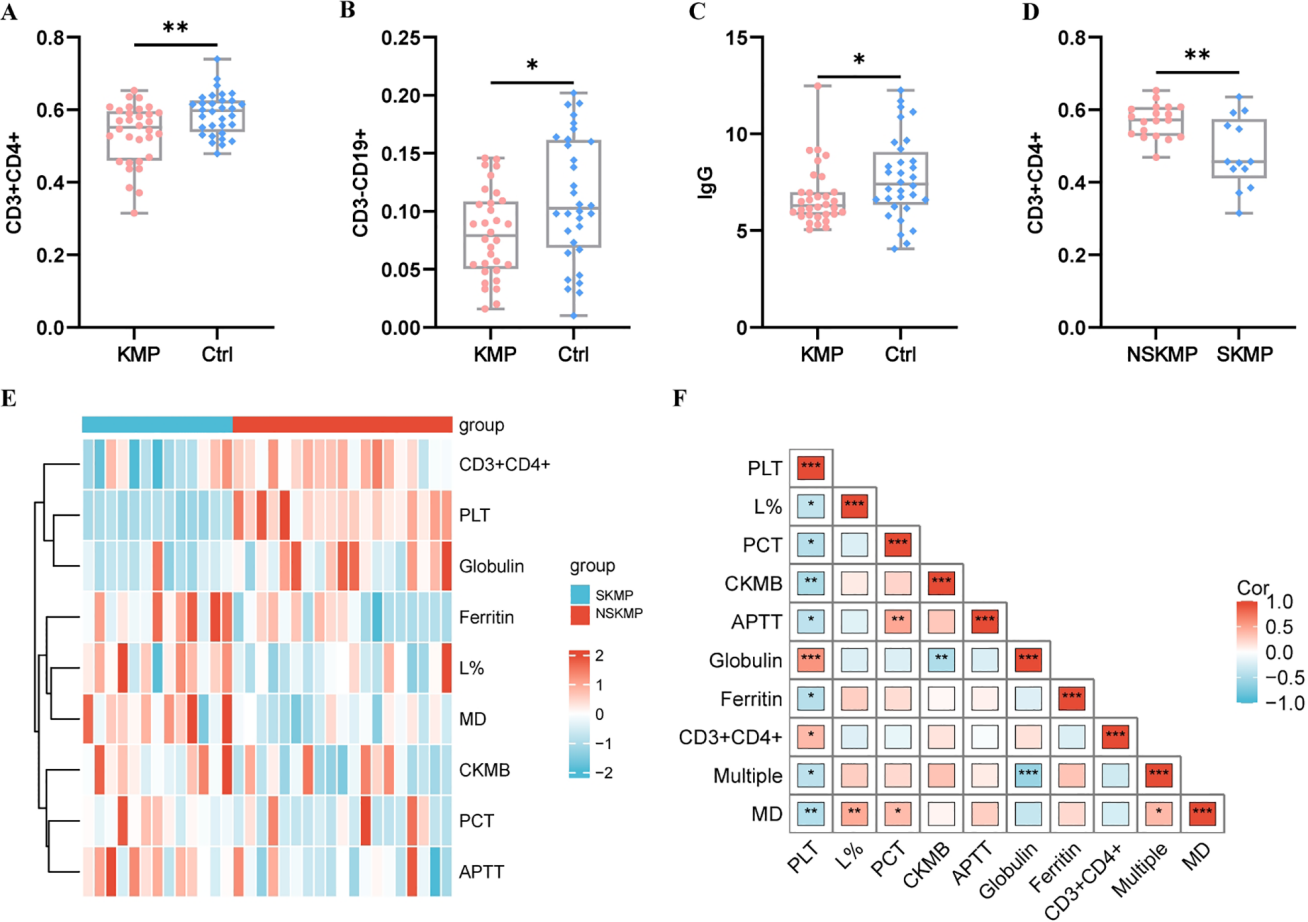
Comparison and correlation analysis of key laboratory parameters. **(A–C)** Comparison of peripheral blood CD3+CD4+ T cells, CD3−CD19+ B cells and IgG levels between healthy controls and KMP patients. **(D)** Comparison of peripheral blood CD3+CD4+ T cells between severe and non-severe thrombocytopenia groups in KMP patients. **(E)** Heatmap showing differences in laboratory parameters between severe and non-severe thrombocytopenia groups in KMP patients. **(F)** Correlation network heatmap of key laboratory parameters in KMP patients. *P < 0.05; **P < 0.01; ***P < 0.001. KMP, Kasabach–Merritt phenomenon; CD, cluster of differentiation; IgG, immunoglobulin G.

The lymphocyte subsets and complement levels of neonates with KMP and the control group are detailed in **Table 1** and **Figure 1**. Lymphocyte subset analysis showed that the proportion of CD3^+^CD4^+^ T cells was significantly lower in the KMP group compared to the control group (0.53 ± 0.08 vs. 0.59 ± 0.06), and the proportion of CD3−CD19+ B cells was also significantly decreased (0.08 ± 0.04 vs. 0.11 ± 0.05) (*P <* 0.05). No significant differences were found between the two groups in the proportions of CD3^+^, CD3^+^CD8^+^, CD4^+^/CD8^+^, and CD3^−^CD16^+^56^+^ cells (*P* > 0.05). Regarding complement and humoral immune markers, the KMP group had significantly lower IgG levels compared to the control group (*P <* 0.05), while no significant differences were found in C3, C4, IgA, and IgM levels (*P* > 0.05).

### 3.2 Baseline and KHE tumor characteristics of severe and non-severe KMP neonates

The baseline and KHE Tumor characteristics of neonates with KMP are detailed in **Table 2**. Among the KMP patients, 13 (40.63%) were in the severe group and 19 (59.37%) were in the non-severe group. The birth weight was 3273.00 (2779.25–3676.00) g, with a median gestational age of 38.57 (37.71–39.71) weeks. Ten patients (31.25%) were delivered by cesarean section. The median age at admission was 17.50 (9.00–24.00) days. Regarding KHE Tumor characteristics, 24 (75.00%) patients had superficial+mixed-type KHE lesions, 8 (25.00%) had deep-type KHE lesions, and 2 (6.25%) had multifocal lesions. Tumor color was red in 8 (25.00%) cases, purplish in 7 (21.88%) cases, and deep purple/ecchymotic in 3 (9.38%) cases. The maximum diameter was 68.50 (52.50–87.25) mm. The lowest platelet count was 41.50 (17.25–48.75) × 10^9^/L.

**Table 2:** Demographic and KHE/vascular tumor characteristics of severe and non-severe KMP patients.

Variable	Total (n = 32)	Severe group (n = 13)	Non-severe group (n = 19)	*P*-value
Baseline characteristics				
Male, n (%)	15 (46.88%)	6 (46.15%)	9 (47.27%)	1.000
Birth weight, g	3273.00 (2779.25-3676.00)	3273.00 (2953.00-3650.00)	3273.00 (2772.50-3765.00)	0.818
Gestational age, weeks	38.57 (37.71-39.71)	38.29 (37.71-39.57)	38.71 (37.93-39.71)	0.991
Caesarean section, n (%)	10 (31.25%)	3 (23.10%)	7 (36.84%)	0.467
KHE tumor characteristics				
Deep, n (%)	8 (25.00%)	4 (30.77%)	4 (21.05%)	0.835
Superficial + mixed, n (%)	24 (75.00%)	9 (69.23%)	15 (78.95%)	0.835
Multifocal, n (%)	2 (6.25%)	1 (7.69%)	1 (5.26%)	1.000
Red colour, n (%)	8 (25.00%)	3 (23.10%)	5 (26.32%)	1.000
Purplish colour, n (%)	7 (21.88%)	4 (30.77%)	3 (15.79%)	0.404
deep purple/ecchymotic, n (%)	3 (9.38%)	1 (7.69%)	2 (10.53%)	1.000
Maximum diameter, mm	68.50 (52.50-87.25)	89.00 (67.00-115.00)	60.00 (46.00-72.00)	0.003
Platelet count, ×10^9^/L	41.50 (17.25-48.75)	16.00 (13.00-18.00)	48.00 (42.00-56.00)	—

KMP, Kasabach–Merritt phenomenon; PLT, platelet count.

The baseline and KHE tumor characteristics of severe and non-severe neonates with KMP are detailed in **Table 2**. No significant differences were observed between the two groups in terms of gender, birth weight, gestational age, and delivery method (*P* > 0.05). The severe KMP group had a significantly larger maximum tumor diameter (*P* < 0.05). There were no significant differences between the two groups in the proportions of superficial+mixed-type KHE lesions, deep-type KHE lesions, or tumor color (*P* > 0.05). , .

### 3.3 Lymphocyte subsets and complement levels in severe KMP neonates

The lymphocyte subsets and complement levels in severe and non-severe neonates with KMP are detailed in **Table 3**. Compared to the non-severe group, the severe KMP group had a significantly lower proportion of CD3+CD4+ T cells (0.48 ± 0.10 vs. 0.57 ± 0.05, *P* < 0.05). No significant differences were observed between the two groups in terms of CD3^+^, CD3^+^CD8^+^, CD4^+^/CD8^+^, CD3^−^CD19^+^, CD3^−^CD16^+^56^+^, C3, C4, IgA, IgG, and IgM levels (*P* > 0.05).

**Table 3:** Lymphocyte subsets and complement levels of severe and non-severe KMP patients.

Variable	Severe group (n = 13)	Non-severe group (n = 19)	*P*-value
Lymphocyte subsets			
CD3+	0.80 ± 0.07	0.79 ± 0.06	0.534
CD3+CD4+	0.48 ± 0.10	0.57 ± 0.05	0.007
CD3+CD8+	0.19 ± 0.02	0.17 ± 0.03	0.054
CD4+/CD8+	2.82 ± 0.66	2.98 ± 0.75	0.521
CD3-CD19+	0.09 ± 0.04	0.07 ± 0.04	0.224
CD3-CD(16 + 56)+	0.09 ± 0.06	0.13 ± 0.06	0.123
Complement and humoral immunity			
C3, g/L	0.67 ± 0.08	0.73 ± 0.25	0.288
C4, g/L	0.17 ± 0.06	0.18 ± 0.08	0.822
IgA, g/L	0.05 ± 0.03	0.05 ± 0.02	0.613
IgG, g/L	6.35 ± 1.11	7.04 ± 1.75	0.184
IgM, g/L	0.30 ± 0.13	0.27 ± 0.14	0.483

KMP, Kasabach–Merritt phenomenon; C3, complement component 3; C4, complement component 4; IgA, immunoglobulin A; IgG, immunoglobulin G; IgM, immunoglobulin.

### 3.4 Comparison of admission laboratory findings between the two groups

**Figure 2:**
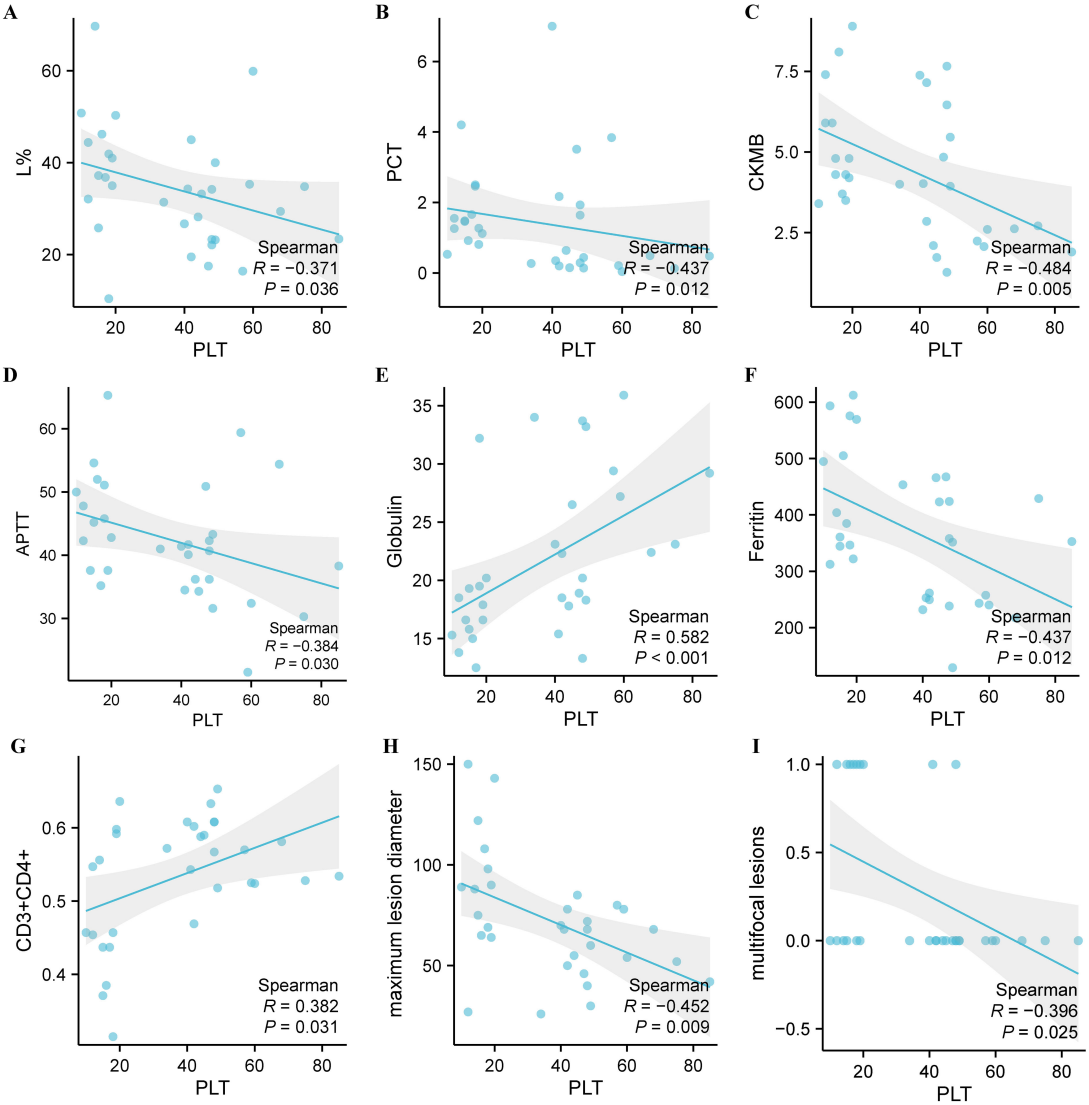
Spearman correlation analysis between platelet count and key laboratory parameters in KMP patients. Scatter plots show the correlations between PLT and L% **(A)**, PCT **(B)**, CKMB **(C)**, APTT **(D)**, globulin **(E)**, ferritin **(F)**, CD3+CD4+ T-cell proportion **(G)**, maximum lesion diameter **(H)**, and multifocal lesions **(I)**. KMP, Kasabach–Merritt phenomenon; PLT, platelet count; L%, lymphocyte percentage; PCT, procalcitonin; CKMB, creatine kinase-MB; APTT, activated partial thromboplastin time; CD, cluster of differentiation.

A detailed comparison of the admission laboratory findings between the two groups is shown in **Table 4**. Compared to the non-severe group, the severe Kasabach-Merritt phenomenon (KMP) group had significantly higher levels of L%, procalcitonin (PCT), creatine kinase-MB (CK-MB), activated partial thromboplastin time (APTT), and ferritin, while the globulin level was significantly lower (*P* < 0.05). No significant differences were observed between the two groups in terms of white blood cell count (WBC), hemoglobin (Hb), neutrophil percentage (N%), monocyte percentage, eosinophil percentage, basophil percentage, reticulocyte percentage (Ret%), C-reactive protein (CRP), high-sensitivity troponin, prothrombin time (PT), fibrinogen, D-dimer, international normalized ratio (INR), total protein, albumin, total bilirubin, direct bilirubin, alanine aminotransferase (ALT), aspartate aminotransferase (AST), creatinine, and urea nitrogen (*P* > 0.05). Spearman correlation analysis showed that PLT was negatively correlated with L%, PCT, CKMB, APTT, ferritin, maximum lesion diameter, and multifocal lesions, whereas positive correlations were observed with globulin and CD3+CD4+ T-cell proportion (**Figure 2**).

**Table 4:** Laboratory findings in severe and non-severe KMP patients.

Variable	Severe group (n = 13)	Non-severe group (n = 19)	*P*-value
Haematological and inflammatory parameters			
WBC, ×10^9^/L	11.07 ± 3.64	10.56 ± 2.89	0.677
Hb, g/L	128.00 (102.00-140.00)	115.00 (96.00-126.00)	0.150
Neutrophils, %	52.98± 13.69	58.61± 10.57	0.199
Lymphocytes, %	40.12± 13.99	30.41± 10.48	0.032
Monocytes, %	8.77 ± 3.78	8.46 ± 2.52	0.801
Eosinophils, %	1.85 ± 1.76	2.37 ± 1.15	0.359
Basophils, %	0.30 (0.20-0.50)	0.40 (0.20-0.65)	0.671
Reticulocytes, %	2.28 ± 1.07	1.63 ± 1.38	0.160
CRP, mg/L	8.59 (4.27-16.35)	6.90 (4.45-18.15)	0.823
PCT, ng/ml	1.46 (1.12-1.66)	0.44 (0.21-1.78)	0.030
Myocardial injury markers			
CK-MB, ng/ml	5.32± 1.80	3.84 ± 2.07	0.045
High-sensitivity troponin, pg/ml	14.23 (0.07-35.63)	19.00 (12.69-23.10)	0.701
Coagulation parameters			
PT, s	16.60 (13.20-22.80)	16.10 (13.75-19.60)	0.514
APTT, s	40.80 (35.95-50.55)	36.10 (32.30-46.30)	0.026
Fibrinogen, g/L	0.72 (0.63-0.98)	0.96 (0.80-1.20)	0.068
D-dimer, mg/L	6.90 (4.80-13.10)	6.68 (4.65- 13.05)	0.822
INR	1.56 ± 0.34	1.57 ± 0.37	0.937
Biochemical parameters			
Total protein, g/L	54.57 ± 6.39	59.38 ± 7.42	0.060
Albumin, g/L	36.63 ± 3.60	35.04 ± 4.94	0.301
Globulin, g/L	17.94 ± 4.86	24.34 ± 6.78	0.004
Total bilirubin, μmol/L	80.85 ± 25.35	57.95 ± 41.99	0.065
Direct bilirubin, μmol/L	17.40 (13.35-25.35)	16.90 (10.65-27.95)	0.897
ALT, U/L	38.40 (26.30-59.90)	66.80 (32.20-94.25)	0.179
AST, U/L	59.00 (43.00-81.10)	72.70 (55.70-93.10)	0.337
Creatinine, μmol/L	38.72 ± 23.23	36.99 ± 10.44	0.805
Urea, mmol/L	4.18 ± 2.62	4.44 ± 1.29	0.749
Ferritin, pmol/L	448.12 ± 113.11	318.21 ± 102.28	0.003

KMP, Kasabach–Merritt phenomenon; WBC, white blood cell count; Hb, haemoglobin; CRP, C-reactive protein; PCT, procalcitonin; CK-MB, creatine kinase-MB; PT, prothrombin time; APTT, activated partial thromboplastin time; INR, international normalized ratio; ALT, alanine aminotransferase; AST, aspartate aminotransferase.

### 3.5 Treatment and short-term outcomes

Treatment and short-term outcomes for neonates with KMP are detailed in **Table 5**. Among the 32 neonates with KMP, 25 (78.1%) received platelet transfusion and fibrinogen supplementation, 16 (50.0%) received plasma transfusion, 14 (43.8%) received intravenous immunoglobulin, and 10 (31.3%) received red blood cell transfusion as non-specific treatments. Regarding specific treatments, 29 patients (90.6%) received corticosteroid therapy, followed by vincristine (19 patients, 59.4%), sirolimus (11 patients, 34.4%), and surgery/interventional radiology (3 patients, 9.4%). Treatment evaluation showed that 25 patients (78.1%) achieved complete remission, 4 (12.5%) achieved partial remission, and 3 (9.3%) had no remission. Among the 25 neonates who achieved complete remission, the platelet count returned to the normal range at a mean of 15 (range: 7 - 35) days after treatment initiation.

**Table 5:** Treatment and outcomes in KMP patients.

Variable	Total (n = 32)	Severe group (n = 13)	Non-severe group (n = 19)	*P*-value
Non-specific treatments				
Intravenous immunoglobulin, n (%)	14 (43.8%)	6 (46.2%)	8 (42.1%)	1.000
Platelet transfusion, n (%)	25 (78.1%)	13 (100.0%)	12 (63.2%)	0.025
Plasma transfusion, n (%)	16 (50.0%)	9 (69.2%)	7 (36.8%)	0.149
Fibrinogen supplementation, n (%)	25 (78.1%)	10 (76.9%)	15 (78.9%)	1.000
Red blood cell transfusion, n (%)	10 (31.3%)	6 (46.2%)	4 (21.1%)	0.244
Specific treatments				
Corticosteroids, n (%)	29 (90.6%)	12 (92.3%)	17 (89.5%)	1.000
Vincristine, n (%)	19 (59.4%)	7 (53.8%)	12 (63.2%)	0.873
Sirolimus, n (%)	11 (34.4%)	5 (38.5%)	6 (31.6%)	0.687
Surgery/IR, n (%)	3 (9.4%)	1 (7.7%)	2 (10.5%)	1.000
Multiple lines of treatment, n (%)	13 (40.6%)	7 (53.8%)	6 (31.6%)	0.208
Outcomes				
Complete remission, n (%)	25 (78.1%)	9 (69.2%)	16 (84.2%)	1.000
Partial remission, n (%)	4 (12.5%)	2 (15.4%)	2 (10.5%)	1.000
No remission, n (%)	3 (9.3%)	2 (15.4%)	1 (5.3%)	1.000
Length of hospital stay, days		19.0 (7.00, 20.00)	18.00 (11.50, 29.00)	0.430

KMP, Kasabach–Merritt phenomenon; IR, interventional radiology. Individual patients frequently received more than one treatment modality, either concurrently or sequentially; therefore, the sum of patients across non-specific and specific treatment categories exceeds the total cohort size (n = 32). Each row represents the number and proportion of patients who received the specified intervention at any point during management. Between-group comparisons (severe vs. non-severe) were performed per treatment modality.

Regarding treatment measures, no significant differences were observed between the severe and non-severe groups in terms of corticosteroids, intravenous immunoglobulin, plasma transfusion, fibrinogen supplementation, red blood cell transfusion, vincristine, sirolimus, multiple lines of treatment, prognosis, and length of hospital stay (*P* > 0.05). However, the platelet transfusion rate was significantly higher in the severe group compared to the non-severe group (*P* < 0.05). Neonates receiving multiple lines of treatment had a higher serum ferritin level (median [IQR]: 404.00 [252.88, 466.00] pmol/L) than those receiving single-line treatment (344.40 [250.34, 426.15] pmol/L), but the difference was not statistically significant (P = 0.393).

### 3.6 Independent risk factors for severe thrombocytopenia in KMP and ROC curve construction

Univariate and multivariate logistic regression analyses of severe thrombocytopenia in KMP are detailed in **Table 6**. To assess the factors associated with severe thrombocytopenia in neonates with KMP, multivariate logistic regression analysis was conducted based on laboratory markers with significant differences in univariate analysis. Due to multicollinearity between CK-MB, PCT, and CD3^+^CD4^+^ and other variables, these three variables were excluded from the subsequent analysis. Ultimately, L%, APTT, globulin, and ferritin were included in the regression model. The results indicated that prolonged APTT (OR = 1.060, 95% CI: 1.010–1.120, *P* = 0.021) and elevated ferritin (OR = 1.010, 95% CI: 1.000–1.020, *P* = 0.048) were independent factors associated with severe thrombocytopenia in KMP.

**Table 6:** Univariate and multivariate logistic regression analyses of severe thrombocytopenia in KMP.

Variable	OR(95% CI) Univariate analysis	P value	OR(95% CI) Multivariate analysis	P value
L%	1.224 (1.030 – 1.454)	0.050	1.268 (0.912 – 1.764)	0.156
CKMB	—	—	—	—
PCT	—	—	—	—
Globulin	0.931 (0.868–1.000)	0.022	0.725 (0.465–1.131)	0.159
Ferritin	1.069 (1.006 – 1.117)	0.009	1.010 (1.000–1.020)	0.048
APTT	1.082 (1.023 – 1.195)	0.040	1.060 (1.010–1.120)	0.021
CD3+CD4+	—	—	—	—

KMP, Kasabach–Merritt phenomenon; OR, odds ratio; CI, confidence interval; L%, lymphocytes percentage; APTT, activated partial thromboplastin time; CK-MB, creatine kinase-MB; PCT, procalcitonin. Variables with collinearity (CK-MB, PCT and CD3+CD4+) were excluded from the multivariate model.

**Figure 3:**
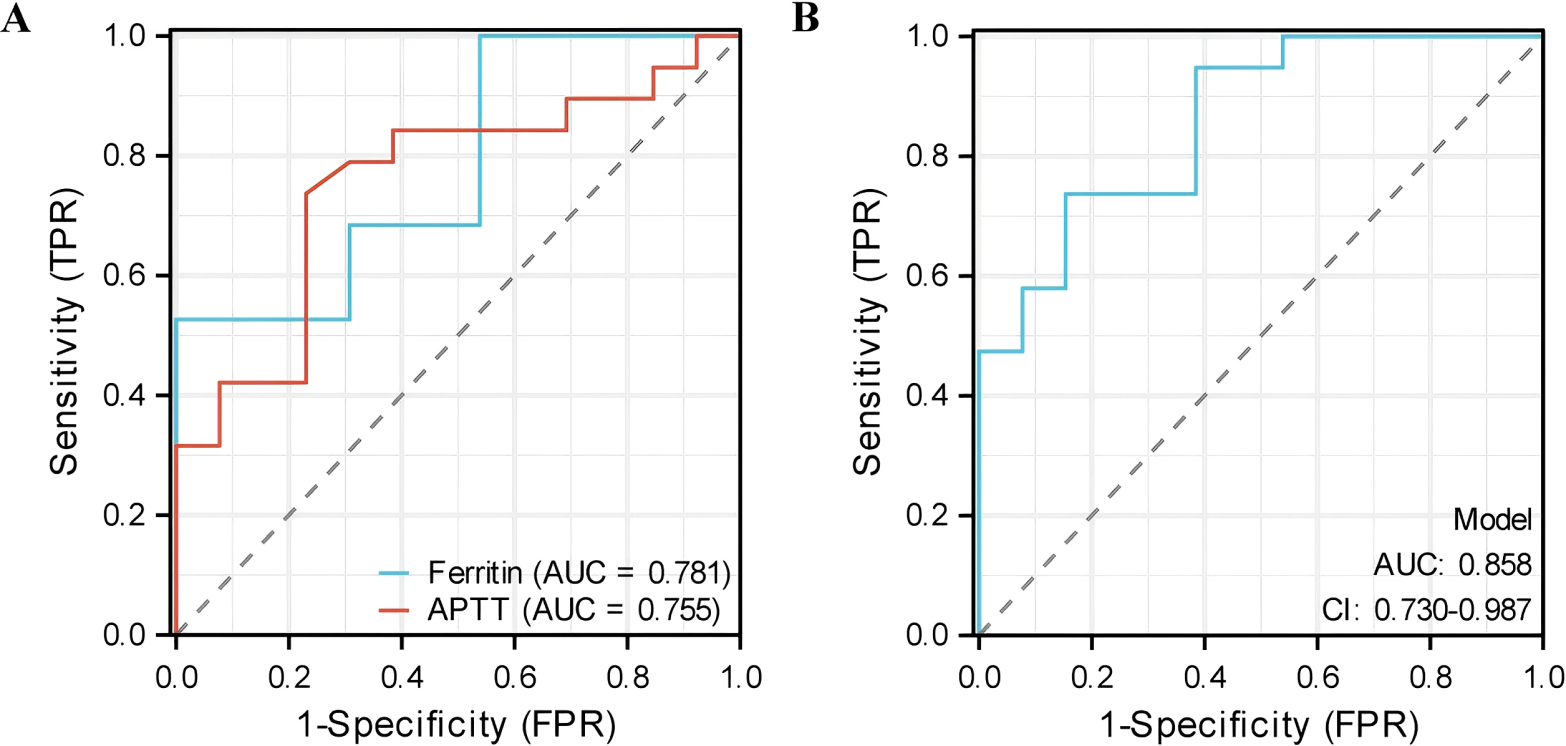
ROC analysis of key predictors for severe thrombocytopenia in KMP patients. **(A)** ROC curves of APTT and ferritin for predicting severe thrombocytopenia in KMP patients. **(B)** Combined ROC curve of APTT and ferritin for predicting severe thrombocytopenia in KMP patients. KMP, Kasabach–Merritt phenomenon; ROC, receiver operating characteristic; APTT, activated partial thromboplastin time.

**Table 7** and **Figure 3** presents the ROC analysis of key predictors for severe thrombocytopenia in KMP. The AUC for APTT was 0.755 (95% CI: 0.580–0.930) with an optimal cutoff of 44.00 seconds, showing a sensitivity of 0.733 and specificity of 0.769. For ferritin, the AUC was 0.781 (95% CI: 0.618–0.945) with an optimal cutoff of 286.85 pmol/L, demonstrating a sensitivity of 0.526 and perfect specificity of 1.000. The combined APTT and ferritin model demonstrated a higher AUC of 0.858 (95% CI: 0.730–0.987), with sensitivity of 0.737 and specificity of 0.846.

**Table 7:** ROC analysis of key predictors for severe thrombocytopenia in KMP.

Variable/model	AUC	AUC (95% CI)	Optimal cutoff	Sensitivity	Specificity
APTT, s	0.755	0.580 – 0.930	44.00	0.733	0.769
Ferritin, pmol/L	0.781	0.618 – 0.945	286.85	0.526	1.000
APTT+Ferritin	0.858	0.730 – 0.987	—	0.737	0.846

KMP, Kasabach–Merritt phenomenon; ROC, receiver operating characteristic; AUC, area under the curve; CI, confidence interval; APTT, activated partial thromboplastin time.

## 4 Discussion

This study systematically reveals the characteristic alterations in the immune system of neonates with KMP, particularly abnormalities in lymphocyte subsets. Compared to healthy controls, neonates in the KMP group showed significantly reduced proportions of CD3^+^CD4^+^ T cells and CD3^−^CD19^+^ B cells, suggesting impairment in both T cell helper function and B cell-mediated humoral immunity. In normal immune responses, CD3^+^CD4^+^ T cells regulate the function of effector T cells and B cells by secreting cytokines such as IL-2 and IFN-γ. Their reduction may lead to a decrease in immune response efficiency and weakened immune regulation, impairing the body’s ability to control infections and pathological signals (6, 15). Additionally, the weakening of B cell function and the reduction in antibody levels (e.g., IgG) may result in a diminished capacity for antibody-mediated immune clearance, making the body more vulnerable in responding to persistent inflammation and vascular tumors (16, 17). Notably, elevated ferritin, together with these lymphocyte and IgG changes, is consistent with observations of immune system activation in pediatric KMP (12) and immune alterations reported in related vascular tumor conditions (9), highlighting a potential role of immune activation in disease pathophysiology.

KMP is not only a consumptive coagulopathy caused by the capture and consumption of platelets and coagulation factors within vascular tumors, but also accompanied by tumor-related immune imbalance and inflammatory responses (8). Previous studies have shown that the interaction between endothelial cells and platelets not only promotes platelet aggregation and consumption but may also amplify the local inflammatory response, further affecting the dynamic regulation of immune cells (18, 19). Prolonged APTT and elevated ferritin were identified as independent risk factors for severe thrombocytopenia, supporting the notion that coagulation abnormalities and inflammation-mediated immune activation jointly contribute to disease severity (12, 20). These interactions can exacerbate coagulopathy, platelet consumption, and tumor progression, forming a vicious cycle. Overall, immune abnormalities in KMP are closely linked to both platelet depletion and coagulation dysfunction, emphasizing the need to consider immune-coagulation interplay when evaluating disease severity and guiding clinical management.

APTT, as a routine coagulation function test, is typically prolonged in the presence of coagulation factor abnormalities. In this study, prolonged APTT was identified as one of the independent risk factors for severe thrombocytopenia, reflecting the close relationship between coagulation factor consumption and platelet depletion. In the vascular tumors of KMP, high levels of angiogenesis and platelet capture enable the tumor tissue to activate local coagulation cascades, thereby accelerating the consumption of coagulation factors and platelets. This process promotes platelet aggregation and microthrombus formation, further consuming coagulation factors and leading to prolonged APTT (3, 8).

Additionally, excessive consumption of coagulation factors may activate the fibrinolytic system, triggering microangiopathic hemolytic anemia, a phenomenon commonly observed in KMP patients with severe thrombocytopenia (13, 21). Therefore, prolonged APTT not only reflects the consumption of coagulation factors but also suggests ongoing endothelial damage and the formation of local microthrombi, further exacerbating platelet depletion and creating a vicious cycle. The formation of these microthrombi and the consumption of coagulation factors not only worsen the bleeding tendency but may also lead to more severe coagulopathy, ultimately affecting the clinical prognosis of KMP. Thus, prolonged APTT can serve as an important indicator for assessing the bleeding risk in neonates with severe thrombocytopenia.

Ferritin is the primary iron storage protein in the body and also a typical acute-phase response protein. Its synthesis can be upregulated by pro-inflammatory cytokines, leading to a significant increase during inflammation and immune activation (22–24). In this study, elevated ferritin was identified as an independent risk factor for severe thrombocytopenia in neonates with KMP, reflecting both acute inflammatory responses and ongoing immune activation. Under inflammation and immune stimulation, macrophages and other immune effector cells are both a major source of ferritin and capable of releasing pro-inflammatory cytokines, enhancing immune responses and the inflammatory cascade, which may contribute to platelet consumption (25, 26). Furthermore, there is a bidirectional regulation between iron metabolism and immune function: changes in iron levels can affect the activity and differentiation of immune cells, while immune activation can enhance the inflammatory response by altering iron homeostasis (27). In the context of KMP, the inflammation and immune cell infiltration within the tumor microenvironment may amplify pro-inflammatory signals, resulting in persistently elevated ferritin levels. This sustained activation not only serves as a marker of inflammation but may also promote immune-mediated platelet depletion and coagulation cascade activation, driving the development of severe thrombocytopenia. Therefore, elevated ferritin in KMP patients reflects both the degree of inflammation/immune activation and the intrinsic connection to the platelet-consuming pathological process.

Regarding treatment and clinical outcomes, our findings are broadly consistent with previously reported results in pediatric KMP populations. In our cohort, 78.1% of neonates achieved complete remission, with platelet counts returning to the normal range in a mean of 15 days (range: 7–35 days), which is comparable to remission rates and recovery times reported in previous pediatric studies (3). A substantial proportion (40.6%) required multiple lines of therapy, reflecting the complexity of managing severe thrombocytopenia. These results align with internationally recognized KMP management strategies, in which corticosteroids and vincristine are first-line agents, and sirolimus is increasingly used for refractory cases, typically combined with supportive measures to correct coagulopathy and control tumor progression (21, 28). Plasma transfusion was administered only under strict pre-specified indications, namely active bleeding, platelet count <10 × 10^9^/L, or fibrinogen <0.5 g/L, consistent with the supportive care protocol described by Ji et al. (29). Although current consensus guidelines generally discourage routine plasma transfusion in KMP, as supplemental coagulation factors may further activate platelet trapping within the tumor and exacerbate consumptive coagulopathy, in this neonatal cohort plasma was reserved as rescue therapy for severe coagulopathy rather than for prophylactic use. In addition, because this study included critically ill neonates treated over a long period (2010–2024), plasma transfusion reflected historical practice patterns before the widespread adoption of current consensus recommendations. Variability in treatment sequencing, choice of agents, and patient age may explain differences across cohorts, highlighting the importance of personalized therapy guided by tumor burden and coagulopathy severity.

Although this study provides preliminary insights into the immune function and severe thrombocytopenia in neonates with KMP, several limitations remain. First, the small sample size and retrospective design are significant limitations of this study. The sample size of 32 KMP neonates may be insufficient to fully represent patients with different clinical subtypes and immune phenotypes. Therefore, the generalizability and reproducibility of the findings require validation through larger-scale prospective multicenter studies. Second, this study employed a cross-sectional design and did not provide long-term follow-up data. The lack of data across a temporal dimension limits our ability to observe long-term immune changes in KMP patients. Future studies should incorporate longitudinal data to explore the dynamic changes in immune system alterations and coagulation abnormalities throughout disease progression and their predictive value for clinical outcomes. Additionally, this study primarily focused on the analysis of static immune markers and did not address more complex immune cell functions or dynamic biomarkers of immune activation, which could provide more detailed information on immune responses. Future research may combine techniques such as flow cytometry to further explore the functional status of immune cells and the actual effects of immune responses.

## 5 Conclusion

This study investigated the immunological features of neonates with KMP and the clinical characteristics of severe thrombocytopenia in KMP. The results indicate that neonates with KMP exhibit significant immune dysregulation, particularly alterations in lymphocyte subsets and decreased immunoglobulin levels. Most patients achieved complete remission after comprehensive treatment. Additionally, prolonged APTT and elevated ferritin were identified as independent risk factors for severe thrombocytopenia, reflecting the close relationship between coagulation factor consumption and immune activation. Together, immune dysregulation and coagulation dysfunction appear to interact, driving excessive platelet consumption and the development of severe thrombocytopenia. These findings offer new perspectives on the pathophysiology of KMP, especially the interplay between the immune and coagulation systems, and provide theoretical support for clinical diagnosis and treatment. Early identification of biomarkers reflecting immune dysfunction and coagulation abnormalities may help enable more precise, individualized treatment strategies for neonates with KMP in the future.

## Data Availability

The original contributions presented in the study are included in the article/supplementary material. Further inquiries can be directed to the corresponding authors.
